# Evaluating changes to *Ralstonia pickettii* in high-purity water to guide selection of potential calibration materials for online water bioburden analyzers

**DOI:** 10.1007/s10295-019-02192-4

**Published:** 2019-07-25

**Authors:** Kurt D. Benkstein, Sandra M. Da Silva, Nancy J. Lin, Dean C. Ripple

**Affiliations:** 1 Biomolecular Measurement Division, Materials Measurement Laboratory, National Institute of Standards and Technology, Gaithersburg, MD 20899-8362 USA; 2 Biosystems and Biomaterials Division, Materials Measurement Laboratory, National Institute of Standards and Technology, Gaithersburg, MD 20899-8543 USA

**Keywords:** *Ralstonia pickettii*, Online water bioburden analyzers, Autofluorescence, Viability, High-purity water

## Abstract

Online water bioburden analyzers (OWBAs) can provide real-time feedback on viable bacteria in high-purity water (HPW) systems for pharmaceutical manufacturers. To calibrate and validate OWBAs, which detect bacteria using scattered light and bacterial autofluorescence, standards are needed that mimic the characteristics of bacteria in HPW. To guide selection of potential standards, e.g., fluorescent microspheres, a relevant bacterial contaminant, *Ralstonia pickettii*, was characterized for size, count, viability, and autofluorescence after exposure for 24 h to HPW or a nutrient environment. The cells exposed to HPW showed smaller sizes, with lower counts and autofluorescence intensities, but similar spectral features. The cell characteristics are discussed in comparison with a set of fluorescent microspheres, considering factors relevant to OWBAs. These studies suggest that fluorescent microspheres should be relatively small (< 1 µm diameter) and dim, while covering a broad emission range from ≈ (420 to 600) nm to best mimic the representative *R. pickettii*.

**Electronic supplementary material:**

The online version of this article (10.1007/s10295-019-02192-4) contains supplementary material, which is available to authorized users.

## Introduction

Water is a key raw material used in many industries where it is essential to monitor for contamination by living organisms. The presence of viable bacteria in high-purity water (HPW) can be detrimental to final industrial products [11, 14]. For pharmaceutical manufacturers, the classic method for assessing viable bacteria in water, also known as bioburden, involves a sampling and culturing approach that is inherently retrospective. Risk assessment and timely decision making are challenging because of the delay in the results owing to the time needed to culture samples. As a result, there is a need for real-time feedback on counts of viable bacteria in purified water systems to facilitate informed, timely decision making. Newly available online instrumentation is proposed to fulfill this need of real-time bioburden detection [6, 9, 17], with the added potential to detect viable, but nonculturable bacteria that would otherwise be missed by culture-based methods [21]. Online water bioburden analyzers (OWBAs) commonly probe the HPW with a 405 nm laser, which yields detection of bacteria through the combination of bacterial scattered light and endogenous autofluorescence, which is excited by the violet light (observation of scattered light only would suggest non-biological particulate matter) [6]. Calibration and validation of these new instruments requires a standard that mimics the scattering and emission characteristics of viable bacteria in HPW.

Fluorescent microspheres have the potential to serve as a calibration/validation material, similar to their employment in quantitative flow cytometry [26–28]. For applicability and relevance to OWBAs, though, certain parameters may have greater impact given the different measurement needs. For example, with the number of bacteria in HPW systems expected to be quite low, a good understanding of microsphere purity, with respect to non-fluorescing microspheres or other particulate matter, will be important. Additionally, the bacteria themselves are likely to undergo changes with exposure to the HPW environment that may affect how they are detected by OWBAs (with changes in bacterial size affecting light scattering and changes to endogenous fluorophores affecting emission intensity and features). To address these issues, a quick assessment was first made of one population of fluorescent microspheres, looking at their size and fluorescence characteristics to establish a baseline for their comparison to bacterial cells as potential calibration materials. Second, the study examined a relevant bacterial species to better inform the comparison with fluorescent microspheres for matching the bacterial characteristics. Here, *Ralstonia pickettii*, a representative contaminant species that can be found in HPW systems [1, 11, 14], was likewise characterized for size and fluorescence (using conventional benchtop approaches rather than an OWBA to facilitate the morphological and spectroscopic comparisons), as well as for count and viability after 24-h exposure to either a typical culturing environment or a HPW environment. Finally, the observed changes in the bacteria samples with environment are discussed along with their potential impact on selection of fluorescent microspheres as a surrogate for bacteria when calibrating/validating OWBAs.

## Materials and methods

*Ralstonia pickettii* (ATCC 700591) was obtained from American Type Culture Collection (ATCC) [4]. Fluorescent microspheres were acquired from Spherotech (catalog number FP-34505-5-4) [4], with a nominal diameter of 3.2 µm, number concentration (or equivalently, number density) of 10^7^ 1/mL, and a maximum emission intensity at 505 nm. The intensity for the microsphere emission is estimated to lie between (4510 and 7900) equivalent reference fluorophores (fluorescein) [27]. Each microsphere is estimated to produce the equivalent emission intensity to between 4510 and 7900 molecules of fluorescein in solution. Fluorescein was acquired from Acros Organics (catalog number 410620010) [4], and diluted gravimetrically in borate buffer solution (pH ≈ 9.2 to 9.3) for fluorescence tests (see below).

### Cell culture

**Fig. 1:**
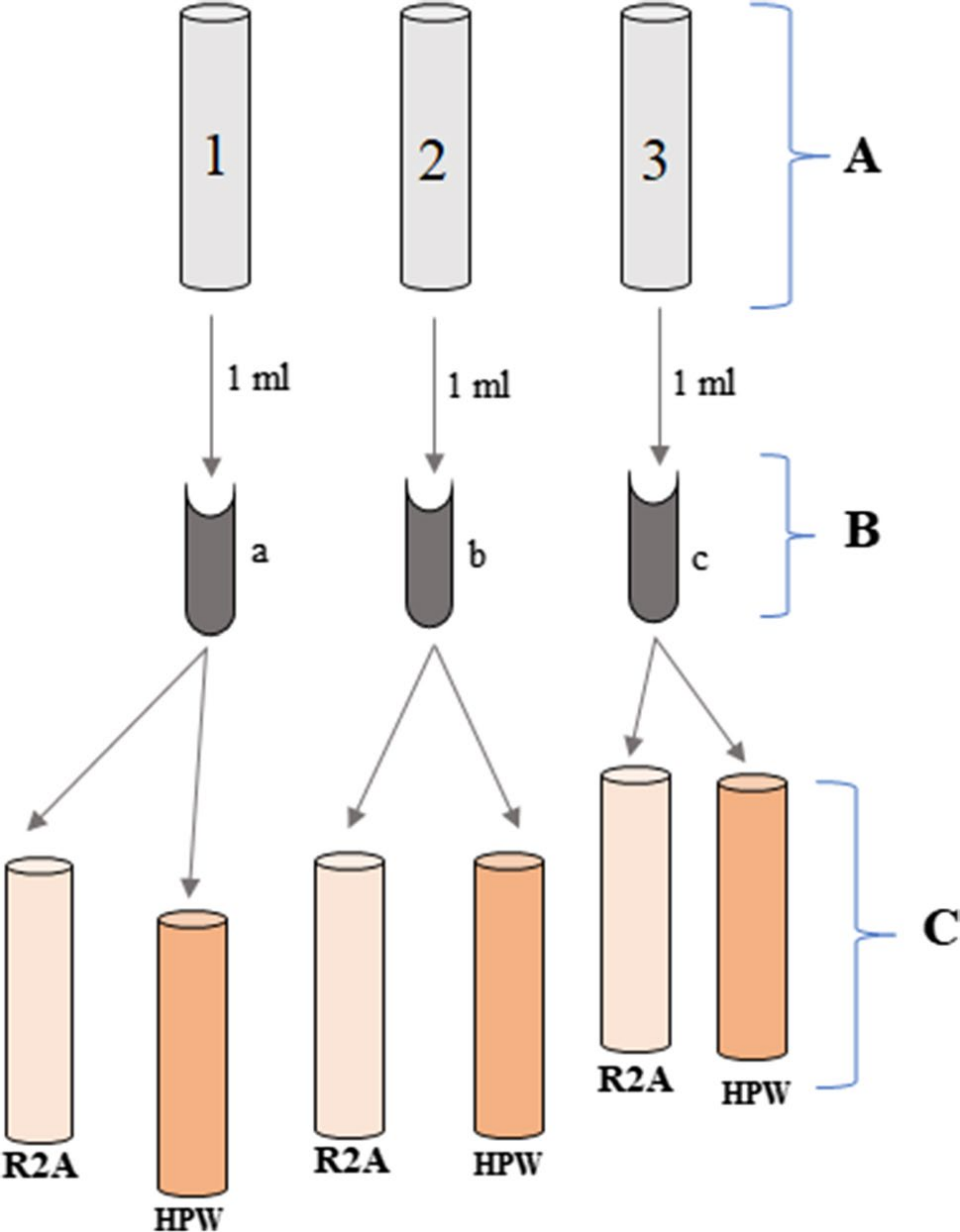
Workflow for preparing samples for HPW and R2A. Samples 1, 2 and 3 (biological replicates) were started independently and grown in R2A broth for 24 h (**A**). One milliliter of each culture was transferred to a new microcentrifuge tube (a–c) and washed twice to remove the broth (rinse step) and determine the initial concentration (**B**). Samples a–c were then diluted in HPW or R2A to prepare the final test samples (**C**)

Cells were prepared by streaking the bacteria onto R2A (Reasoner’s 2A [19]) agar plates and incubating them at 30 °C for 72 h or until colonies were visible. The experimental workflow is shown schematically in Fig. 1 and described in detail here. For each experiment, each of three biological replicates was prepared by inoculating five colonies into 3 mL R2A broth and culturing for 24 h at 30 °C under 150 rpm shaking (Incu-Shaker Mini, Benchmark, Edison, NJ, USA) (Fig. 1a) [4]. From each culture, 1 mL was transferred to a 1.5 mL microcentrifuge tube and washed twice by spinning at 6000×*g* (r.c.f.) for 2 min and re-suspending in 1 mL of sterile HPW (resistivity 18 MΩ cm at 25 °C, autoclaved for 20 min at 121 °C) (Fig. 1b). The number of cells per milliliter was determined by electrical sensing zone analysis as described below. The washed cells were then diluted into 3 mL of the final environment (R2A or HPW) for a final cell number concentration of ≈ 2 × 10^8^ 1/mL, such that each biological replicate generated one sample in HPW and one sample in R2A broth (Fig. 1c). At time zero, the viability of the cells was measured by drop plate method to determine the number of colony forming units (CFU) per volume [10], and the number concentration of cells was determined by electrical sensing zone analysis. The cells were exposed to R2A or HPW for 24 h at 30 °C under 150 rpm and subsequently spun down at 6000×*g* (r.c.f) for 2 min. Pellets were re-dispersed in 3 mL 0.85% sodium chloride by mass, and the viability and number concentration of cells were determined again. At this point, the cells were ready for autofluorescence measurements.

### Viability measurement by plate counting

Cell viability was quantified using a drop plate method [10]. Briefly, each cell suspension was prepared as a tenfold serial dilution in phosphate buffered saline containing 0.04% (by mass) polysorbate 80 (PBST) [4]. Three 10 µL drops per dilution were deposited on R2A agar plates, and plates were incubated at 30 °C for 48 h or until colonies were visible. Only dilutions from 10^4^ to 10^7^ were analyzed. When comparing viability between time *t* = 0 h and *t* = 24 h, statistically significant differences in viability were defined as *P* < 0.05 for the pairwise *t* test.

### Cell number concentration

Cell number concentration was determined by electrical sensing zone analysis. Briefly, 20 μL of cell suspension was dispersed into 20 mL Isoton II and analyzed using a Multisizer 4 (Beckman Coulter, Indianapolis, IN, USA [4]) with the following parameters: 20 µm aperture tube and 100 µL analytical volume. Bacterial size was estimated based on the diameter of an equivalent sphere, as quantified by instrument software.

### *R. pickettii* fluorescence

Fluorescence spectra for the *R. pickettii* cells were measured using a Horiba Fluorolog 3/22 spectrofluorometer [4]. All spectra were measured under identical conditions: *λ*_ex_ = 405 nm, slits at 4 nm, *λ*_em_ = (415–750) nm, 1 nm per step, integration time of 0.2 s per step. A 10 mm glass fluorescence cuvette was used to hold the cell dispersions. For each *R. pickettii* sample, the primary (undiluted) sample and two serial dilutions of that sample were measured to yield a range of cell concentration densities from (≈ 2 × 10^8^ to ≈ 5 × 10^6^) 1/mL. The spectral emission intensities were normalized by dividing the emission intensity (counts per second, cps) by the lamp intensity (mA), as measured by a reference detector, to compensate for any changes in the lamp output over wavelength and from run-to-run. Background spectra were acquired of 0.85% saline solution between each type of cell (R2A vs. HPW). For each sample or background, three spectra were generally acquired. Background spectra did not show additional features over the course of each day, indicating that the cuvette was sufficiently cleaned from the previous sample. For analysis, the averaged background spectrum, which included spectra acquired throughout the day, was subtracted from the sample spectra. For excitation at 405 nm, the water Raman band was a prominent feature in both the background and the sample spectra, with a peak intensity at *λ* ≈ 469 nm. Background-subtracted sample spectra were analyzed for their integrated fluorescent intensity from 491 to 590 nm, which avoided the interfering effects from the water Raman band subtraction. Spectra were also acquired for dispersions of silica particles of diameter *d* = (0.527 ± 0.017) µm or *d* = (1.173 ± 0.064) µm), at a concentration of ≈ 1 × 10^8^ 1/mL to examine the effects of light scattering on the spectral profiles. To estimate equivalent reference fluorophores [27], a calibration curve for fluorescein fluorescence intensity was generated based upon integrated intensity (parameters matched that of the autofluorescence spectra) as a function of fluorescein solution concentration.

### Microsphere characterization

Fluorescent microspheres were characterized for their count, size, morphology, purity and fluorescence using flow-imaging and stop-flow microscopies. The flow-imaging microscope (Micro-Flow Imaging DPA-4200, ProteinSimple [4]) was calibrated for counts using previously calibrated microsphere solutions [20]. The microspheres were characterized at three target concentrations ranging from (≈ 3 × 10^4^ to ≈ 9 × 10^4^) 1/mL.

A custom stop-flow microscopy system was used in both bright-field and fluorescence imaging modes. Microspheres, ≈ 100 total, were imaged after settling to the bottom of a flow cell with flow stopped. Fluorescence images were acquired using a filter cube with 445 nm excitation wavelength and 30 nm bandwidth and emission at 500 nm wavelength and 40 nm bandwidth. Both brightfield and fluorescent microscopy images were analyzed using ImageJ [24]. In brightfield mode, each image was first divided by a background image to correct for variations in illumination intensity. A binary image was created by applying a threshold equal to 96% of the median image intensity. The Analyze Particles feature in ImageJ gave position and area of the resulting particles. The resulting diameters were corrected by subtracting a fixed value so that the mean microsphere diameter obtained was equal to the mean diameter obtained from the commercial flow-imaging microscope, which is calibrated with microspheres of known diameter. Fluorescence intensities of the microspheres were determined by subtracting the background intensity level for each frame, and then integrating fluorescence over a 50 pixel (20.6 µm) diameter circle (chosen to extend the area to where the fluorescence signal became comparable to the background noise level) for each microsphere.

Fluorescence spectra were acquired using *λ*_ex_ = 488 nm, slits at 5 nm, *λ*_em_ = (500–650) nm, 1 nm/step, integration time of 0.8 s/step. A 10 mm glass fluorescence cuvette was used to hold the microsphere dispersion for collection of spectra.

## Results and discussion

### Assessment of fluorescent microspheres for OWBA

Fluorescent microspheres are potential calibration materials for OWBA systems. They are a generally available material with a wide variety of fluorescent dyes that can cover emission wavelength ranges of interest to either side of the water Raman band. They can be well-characterized for intensity, traceable to the SI (International System of Units) [7, 25, 27]. To characterize other parameters that may affect the suitability for application as calibration materials for OWBA systems, several measurement approaches were used. Because of the generally low detection levels associated with OWBA instruments and high-purity water systems, an emphasis was placed on differentiation of fluorescent microspheres from other non-fluorescing particulate matter in the particle dispersion that could be present (e.g., container debris or unlabeled microspheres).

**Fig. 2:**
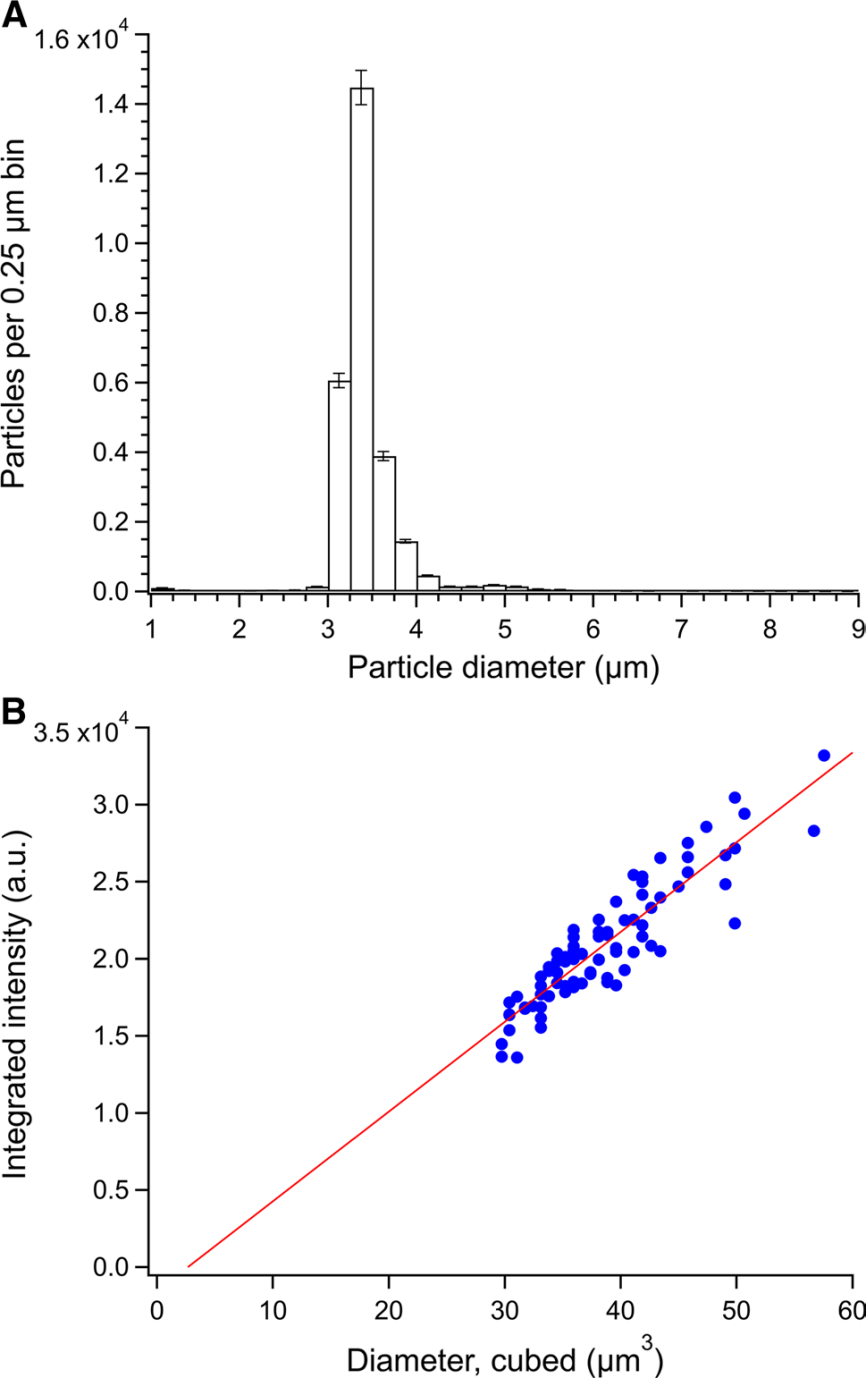
Characterization results for fluorescent microspheres. The particle size distribution (**a**) from flow-imaging microscopy, using 0.25 µm size bins. The error bars represent  × 2 the relative standard uncertainty. The qualitative comparison of fluorescent intensity versus microsphere size (diameter cubed) for *n* = 83 microspheres (**b**) as measured by stop-flow microscopy for single, fluorescent particles. The data are well fit by a linear model (red line), which yields a slope of (582.1 ± 30.5) a.u./µm^3^ and a *y*-intercept of (–1554 ± 1190) a.u.

Figure 2 shows the results of the analyses by flow-imaging microscopy and stop-flow microscopy. Flow-imaging microscopy was employed to study the counts, sizes, and morphologies of particles in the fluorescent microsphere dispersion. The measured particle distribution is shown in Fig. 2a. The peak of the distribution is centered at 3.375 µm. A small peak or shoulder in the histogram is observed just below 5 µm diameter, which is likely associated with doublets of the main population of microspheres. Any doublets or larger aggregates of the main population appear to be no more than ≈ 3% of the total number of particles counted. However, the resolution of the flow-imaging microscope was not sufficient to confirm the presence of particle clustering by resolving them into their component microspheres. For higher resolution assessment of particle morphologies, a stop-flow approach was used, in which the particles were allowed to settle onto a substrate before being imaged. Using this approach, the morphologies of the particles could be more readily assessed. Furthermore, the microscope set-up was equipped with a light source that could excite the dye in the fluorescent microspheres. In this way, the number of fluorescing microspheres could be compared with non-fluorescing particulate matter of similar size and morphology. Figure 2b shows the relative intensity of the fluorescent particles as a function of their diameter cubed. The points are well modeled by a linear fit, which indicates a volumetric dependence of the emission intensity. This is not unexpected given a hard-dyed fluorescent microsphere, that is, one that had dye molecules distributed throughout the entire volume of the sphere. Furthermore, only about 1% of the population in this size regime was non-fluorescent. These methods characterize the fluorescent microspheres for size, count, and purity as a basis for comparing with the bacteria they are meant to mimic. To complete this comparison, a relevant bacterial species was similarly characterized.

### The impact of nutrient levels on *R. pickettii* growth

*Ralstonia pickettii*, Gram-negative and rod-shaped, is a common bacterial contaminant found in oligotrophic environments, including high-purity and ultrapure water supplies used for the semiconductor and pharmaceutical industries, as well as in purified water systems in space vehicles [1, 11, 14, 23]. The survival strategies and biofilm formation of *R. pickettii* in oligotrophic environments have also been featured in several papers [1, 11, 14]. Owing to its ubiquitous presence in HPW systems and study history, *R. pickettii* was selected as an industrially relevant test species, suggested by the OWBA Workgroup (see Acknowledgements), for examining the effects of a low-nutrient water environment on its cell size and autofluorescence characteristics, which may impact detection of bacteria by OWBAs. Previous reports have discussed various strategies for bacteria to cope with starvation conditions [22], including changes to cell size and shape, as well as changes in autofluorescence [2, 21]. These studies on other bacterial species surveyed a range of excitation and emission wavelengths, but tended to emphasize excitation wavelengths less than 400 nm and emission wavelengths less than 460 nm [2, 21]. OWBAs commonly use an excitation wavelength of 405 nm, and can monitor the emission intensity at wavelengths corresponding to emission from nicotinamide adenine dinucleotide (NADH, *λ*_em_ ≈ 450 nm) and flavin molecules (*λ*_em_ ≈ 520 nm) [6, 15, 17].

The survival of *R. pickettii* in these nutrient-depleted environments has been attributed to their formation and residence in biofilms, which can concentrate any residual nutrients present [14]. Changes in bacteria after exposure to high-purity water (e.g., after cells transition from the biofilm environment to a planktonic form [5]) could affect their characteristics that impact detection by OWBAs, and may influence the choice of a calibration material. In this work, *R. pickettii* was exposed for 24 h to either a nutrient environment (R2A growth medium [19]) or an essentially nutrient-free HPW environment to evaluate differences in overall count, size, and autofluorescence spectral features and intensity of the bacteria, focusing on emission from excitation at 405 nm. R2A medium is generally used for the culture of bacteria found in potable water systems [11], but the minimal nutrient R2A is still much more nutrient rich than HPW. The workflow for the bacterial sample preparation is shown in Fig. 1. A series of these experiments was performed over multiple days using independent cultures to prepare the cell samples to explore biological variability with the cell characterization.

**Fig. 3:**
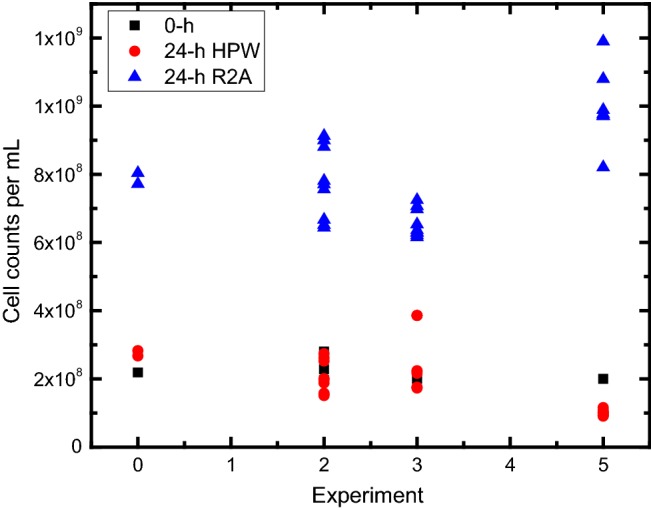
Cell counts concentration (1/mL) measured by electrical sensing zone analysis to compare cell growth between R2A and HPW after *R. pickettii* were exposed to these two conditions for 24 h. The graphic also depicts the cell counts concentration at the zero time. Note that both conditions (HPW and R2A had the same initial cell concentration at 0 h)

**Fig. 4:**
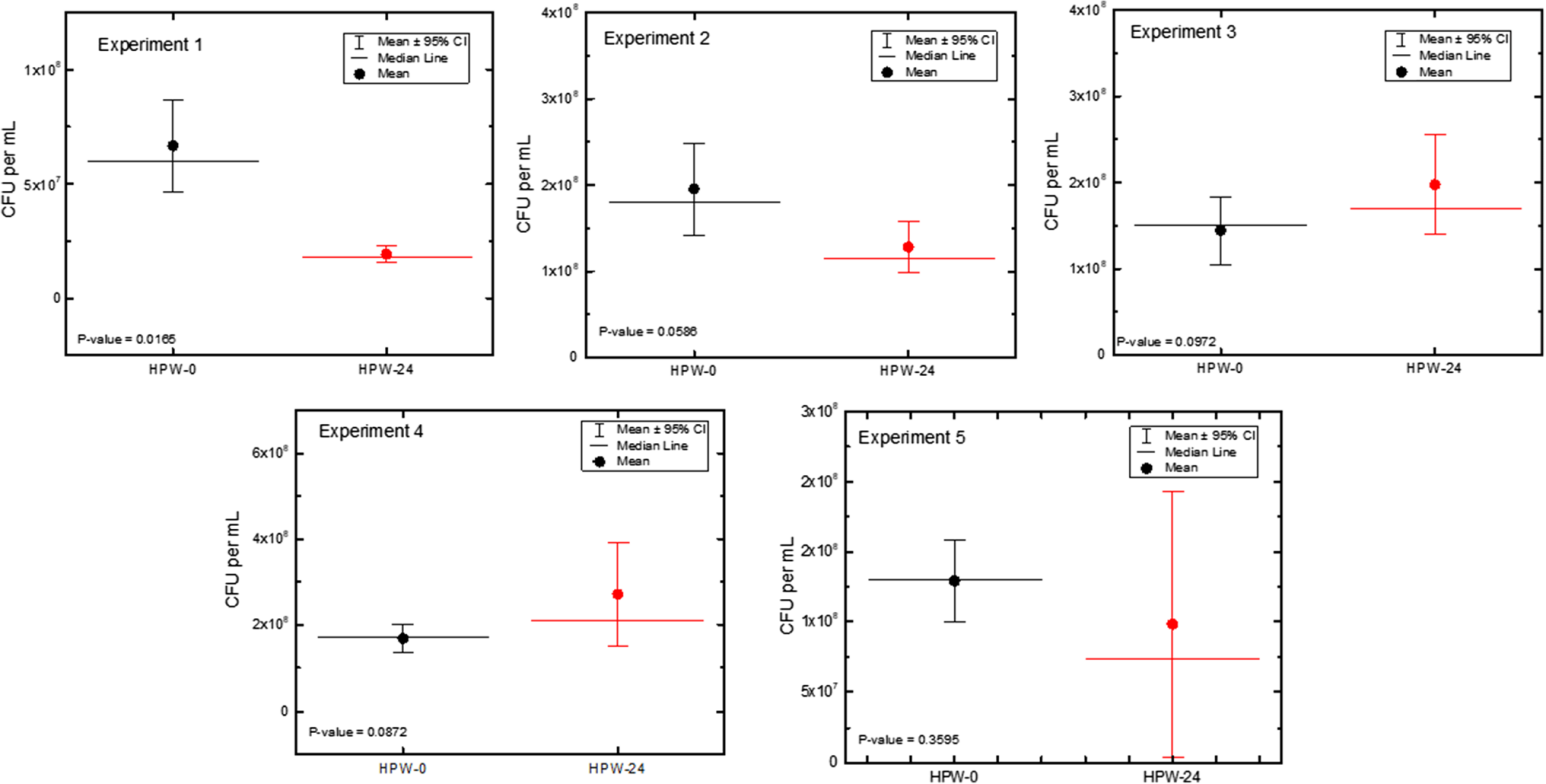
Viability of *R. pickettii* exposed to HPW at two-time points, zero and 24 h for five separate experiments performed on different days (4 ≤ *n* ≤ 9). The data suggest that the cells are still viable after 24 h. Pairwise *t* test indicate that there are statistically significant differences in cell viability only for experiment 1

Figure 3 shows the effects of 24 h of exposure to the two environments on cell counts as measured by electrical sensing zone analysis. Initial cell number concentrations varied slightly from experiment to experiment and ranged from ≈ 9.0 × 10^7^ 1/mL to ≈ 3.9 × 10^8^ 1/mL. After 24 h, no apparent cell growth was observed for the *R. pickettii* in HPW. On the other hand, samples in R2A showed an increase in cell number concentration of ≈ 3.3-fold for 20 out of 28 sample replicates (Fig. 3). Differences in cell diameter between the two conditions were also observed. The average cell diameter after 24 h in R2A was 1.00 µm (SD_data_ = 0.05, *n* = 28), while for 24 h in HPW the cell diameter was 0.71 µm (SD_data_ = 0.07, *n* = 28) (Figure S1). This diameter difference corresponds to a difference in volume of nearly a factor of 3. Cell size reduction in HPW suggests a starvation scenario [1, 22], due to lack of sugar, amino acids and other nutrients relevant for cell growth [29]. To investigate whether the cell-size reduction in HPW was an indication of loss in viability, CFU measurements were made using plate counting as described in Figs. 4 and S2, and discussed statistically in the supplemental material. These tests demonstrated that cells remained viable after 24 h in either environment.

### Characterization of *R. pickettii* autofluorescence

**Fig. 5:**
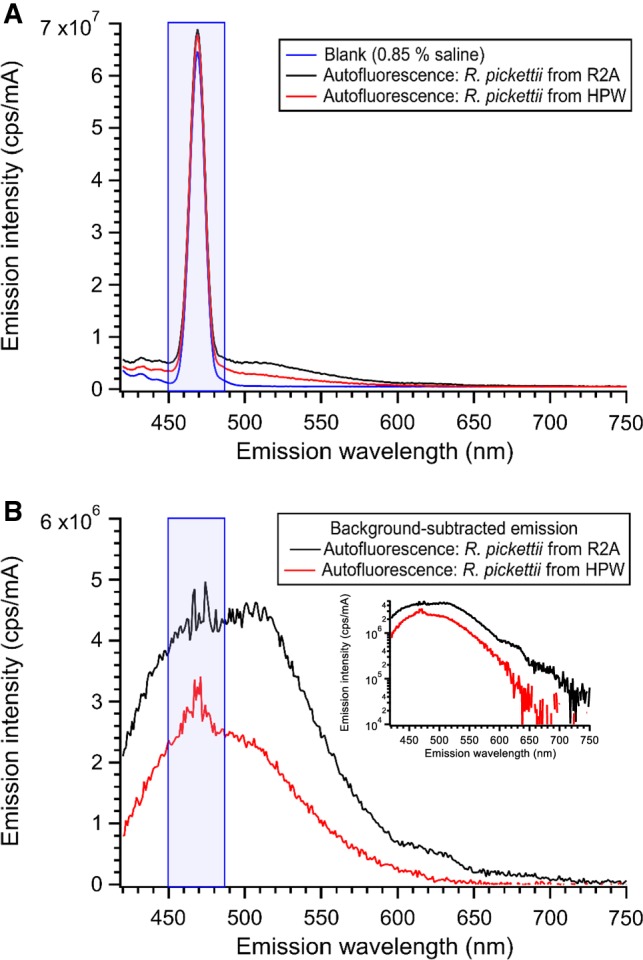
Emission spectra with *λ*_ex_ = 405 nm. The water Raman scattering band is highlighted in blue. **a** Raw emission spectra of a background solution (0.85% saline) and for *R. pickettii* after exposure to R2A or HPW environments for 24 h. **b** Background-subtracted emission spectra from 4a for *R. pickettii* exposed to R2A or HPW. Cell concentration for each is ≈ 9.2 × 10^7^ 1/mL. The inset shows the same spectra on a logarithmic *y*-axis

Cell number concentrations for the autofluorescence measurements generally ranged from ≈ 2 × 10^8^ 1/mL to ≈ 5 × 10^6^ 1/mL. Example spectral profiles for a background spectrum (0.85% saline) and for autofluorescence spectra (*R. pickettii* exposed to R2A and HPW environments) are shown in Fig. 5a. The prominent peak with a maximum intensity at 469 nm is the water Raman scattering band from excitation at 405 nm. The emission to the red of the water Raman band has a peak at ≈ 510 nm and is attributed to flavin molecules in the bacteria [3, 17]. The spectra shown in Fig. 5b are from samples at a similar concentration of *R. pickettii* (≈ 9 × 10^7^ 1/mL) and demonstrate that cells exposed to the HPW environment have reduced emission intensity. The inset to Fig. 5b shows the same spectra on a logarithmic *y*-axis, which can highlight differences in spectral shape. The two samples show generally similar spectral shapes with emission covering a broad range from less than 420 to ≈ (650 to 700) nm. The spectrum from the sample exposed to the R2A environment shows additional emission intensity as a peak/shoulder from ≈ 600 to ≈ 650 nm on the red tail of the main peak. This feature, commonly (but not always) observed in spectra of cells exposed to the R2A environment (see for example Fig. S3), is indicative of additional fluorophores in those samples. The excitation and emission ranges would suggest a porphyrinic fluorophore (see, for example, [18]), although additional work would be needed to positively identify the source of this emission feature. The contributions of scattered light to the fluorescence intensity were also investigated using silica particles (Supplemental Material). The low signal intensity from the scattering indicates that the largest contribution of the measured emission intensity is from the autofluorescence intensity of *R. pickettii*.

**Fig. 6:**
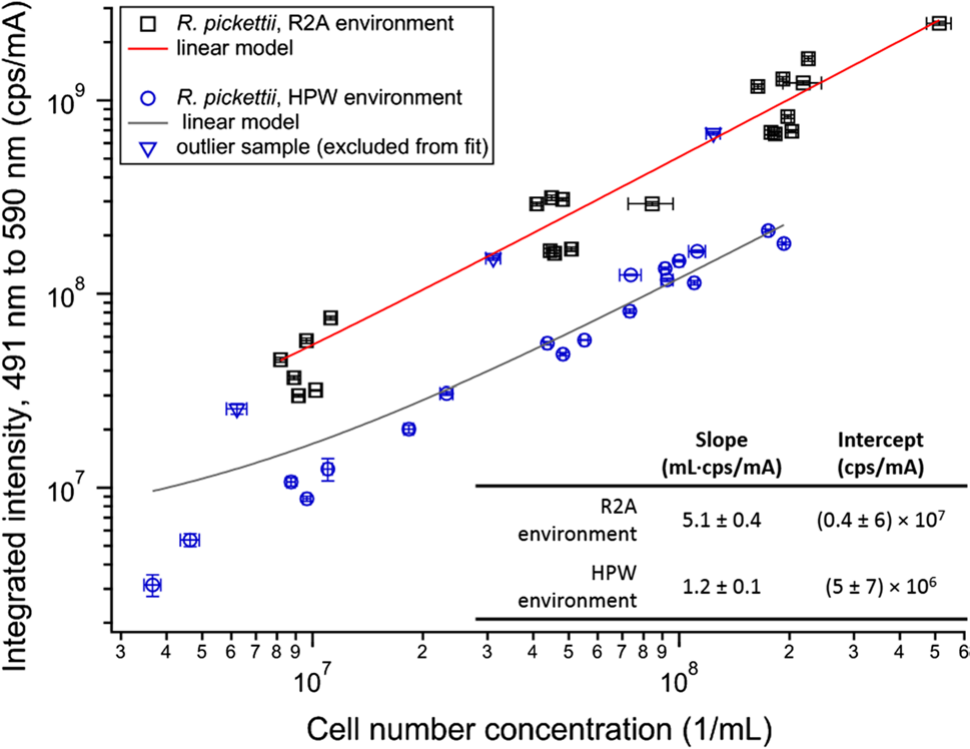
A plot of autofluorescence integrated intensities versus cell concentrations of *R. pickettii* exposed to R2A or HPW environments. Error bars along the *x*-axis reflect the standard deviations of the electrical sensing zone measurements (*n* = 3) and uncertainties associated with the dilutions by pipette. Error bars along the *y*-axis reflect the standard deviations of the integrated intensities (*n* = 3 for the primary samples, or 2 for some replicates). Note that error bars may be obscured by the data markers for relatively small error values

Of particular interest for detection of bacteria in HPW using OWBAs are changes in emission intensity and profile for the *R. pickettii* exposed to the two environments. Figure 6 shows the integrated emission intensities for the cell samples plotted versus their concentrations. Previous studies have followed trends in tryptophan emission [2], but here the interest is in the emission from flavin-based fluorophores at wavelengths greater than the water Raman band. Following the procedures described above, emission spectra were obtained and analyzed for each bacterial sample series over multiple experiments (days). After background subtraction, the resultant emission spectra were analyzed for their integrated intensity from 491 to 590 nm, which mitigates the impact of artifacts from the water Raman background subtraction and other fluorophores that can emit at longer wavelengths. The integrated intensities generally fall into two populations that are dictated by the environment to which the cells were exposed before testing. However, for one experiment there is a set of samples (the primary sample and two dilutions, shown as blue triangles) from the HPW environment that overlaps with the sample sets from the R2A environment. Taking into consideration other data from that sample set, the bacterial size and counts for that sample fit well with the other samples from HPW, suggesting that they were not exposed to additional nutrients (e.g., from incomplete washing of the initial growth medium). Based upon the spectral shape (Fig. S3), which is consistent with the shape of the other HPW samples, particularly for wavelengths greater than ≈ 600 nm, it appears to be a highly fluorescent sample rather than contamination with a sample from the R2A environment. Further studies would be needed to explore the conditions that led to the high emission intensity from that sample. Each group (excluding the highly fluorescent samples from the HPW-environment group) can be fit reasonably well with a linear model, with the *y*-intercepts and slopes shown in Fig. 6. (Note, that the slope for the linear model that includes all points from HPW tests shifts to 1.57 ± 0.45.) The difference in slopes between the two fits indicates that the emission intensities from samples exposed to HPW are a factor of ≈ 4.4 less intense than those exposed to the R2A environment. As noted in the discussion above, the cell volume decreased by a factor of ≈ 2.8 in comparison to cells from R2A to HPW environments, which corresponds to the lower emission intensity for the cells exposed to HPW. These results suggest that bacteria in HPW systems monitored by OWBAs will demonstrate relatively low emission intensities from the flavin-based fluorophores as compared to cells that have a readily available source of nutrients.

### Comparison between *R. pickettii* and the fluorescent microspheres

**Fig. 7:**
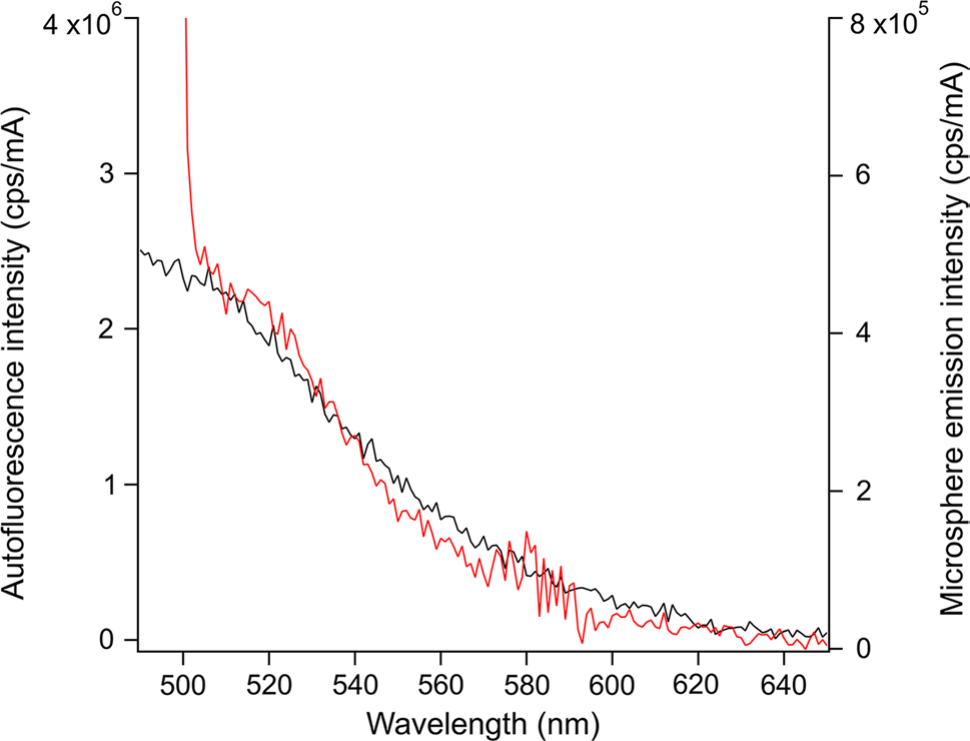
Emission spectra from *R. pickettii* exposed to HPW (black, left axis) and from fluorescent microspheres (red, right axis) comparing the spectral profiles and coverage. The spectra have been normalized to cell or microsphere number concentration, as appropriate. The portion of the spectrum for the microspheres for *λ* < 504 nm is likely an artifact from the relatively wide excitation/emission slits and excitation at *λ* = 488 nm

As noted, the characterization of the changes to *R. pickettii* after exposure to HPW for 24 h is intended to provide a relevant test case for comparing a potential OWBA calibration material. Figure 7 shows an emission spectrum from the fluorescent microspheres (normalized to the microsphere concentration ≈ 1.2 × 10^5^ 1/mL) as excited at *λ* = 488 nm on the right axis, as compared with a typical *R. pickettii* emission spectrum after exposure to a HPW environment (left axis, normalized to cell number concentration, ≈ 9.2 × 10^7^ 1/mL). Because of the relatively low microsphere stock concentration, a matched comparison was not feasible (i.e., same concentration and excitation wavelength). It should also be noted that the cell number concentration is relatively high compared to what might be expected in a HPW system: the higher cell number concentration facilitated the acquisition of the autofluorescence profile. This enabled a qualitative comparison in spectral profiles between the fluorescent microspheres and the bacterial autofluorescence was performed. The emission profile from the microspheres demonstrates similar coverage over the shown wavelength range, which corresponds generally to the integral range used in this study. This wavelength range corresponds to the emission attributed to endogenous flavins. To cover the higher energy emission attributed to endogenous NADH, the use of a second fluorophore-dyed microsphere would need to be targeted to the emission with *λ* < 469 nm.

The intensities for the cells and microspheres may also be compared using the equivalent reference fluorophore (ERF) approach [27]. Because the ERF approach compares the intensities based upon equivalent fluorophores (fluorescein in this case), it is possible to make a quantitative comparison. The values for the cells were estimated based upon a fluorescein calibration curve from spectra generated using the same excitation profile and integral range. The ERF value for the cells exposed to the HPW environment is estimated to be ≈ 2800 fluorescein molecules per cell, while those exposed to the R2A environment are estimated to be ≈ 10,000 fluorescein molecules per cell. In comparison, the microspheres are estimated to have an ERF value of ≈ 6000 fluorescein molecules per microsphere (from the manufacturer), which falls between the ERF values estimated for the cells from the two environments.

Finally, while not a focus of this study, it should be noted that the size and refractive index of the particles (bacterial or fluorescent microspheres) will impact how strongly they scatter light, which affects particle detection in OWBAs. The microspheres studied here are expected to scatter light more strongly than the *R. pickettii* based upon their larger size (≈ 3.4 µm diameter) and refractive index (≈ 1.59, as reported by the manufacturer). *R. pickettii* have sizes smaller than the fluorescent microspheres studied here, with the bacteria having equivalent diameters ≈ 3 times to ≈ 4 times smaller than the microspheres, depending upon their exposure to nutrients. Furthermore, the refractive index for bacterial cells is expected to be relatively low, with reports in literature giving values for the refractive index of various species as ≈ 1.39 [13, 16]. If the sizes of the microspheres and the bacteria were matched, the difference in refractive indices could yield a difference in scattering intensities approaching two orders of magnitude, depending upon the scattering angle (with 405 nm light, 1 µm diameter spherical particle with *σ* = 10%, and refractive indices as above; estimated using MiePlot 4.6) [12].

These results provide some guidance on selection of a calibration material for OWBAs, in particular, for fluorescent microspheres. The study demonstrates that the bacteria will adopt typical starvation strategies after 24 h in HPW that could affect the methods used by OWBAs for bacterial detection. Factors of importance for OWBAs include a decrease in cell size and a reduction in growth rate. Furthermore, the emission intensity from endogenous fluorophores is decreased, while maintaining similar spectral features (i.e., emission from both NADH- and flavin-based fluorophores). To be well matched to the *R. pickettii* from this study, the fluorescent microspheres should be relatively small (*d* < 1 µm) and with relatively low intensity (< 3000 equivalent fluorescein molecules per microsphere). To cover the full range of emission, a second microsphere population (or a microsphere population embedded with two fluorophores) would be required. If feasible, a microsphere material with a lower refractive index might also be considered to lower the intensity of the scattering signal.

Opportunities also exist to expand on the studies described here. With respect to fluorescent microsphere characterization, the stop-flow microscopy may be modified to accommodate smaller fluorescent microspheres and higher throughput (e.g., automation to enable characterization of higher numbers of particles than the ≈ 100 particles characterized for this demonstration). Indeed, continued development of this approach could implement an induced settling to the substrate to decrease the wait time for smaller microspheres, as well as other potential light sources that can be used to excite different fluorophores. To establish a microsphere as a reference material would involve a similar process to what is currently used in the flow cytometry community [28]. Successful application of a reference material would require working with end-users to ensure relevancy to their needs. Opportunities also exist for extending this study to look at additional relevant species and effects on bacterial cell properties of longer-duration exposures to HPW, perhaps corresponding to timeframes commonly used between water-loop sterilization cycles. Furthermore, a more exhaustive analysis of changes in endogenous fluorophore populations resulting from exposure to low nutrient environments could address some of the outstanding questions related, for example, to the loss of emission peak at ≈ 620 nm commonly observed in cells exposed to the R2A medium, or to the relatively high intensity emission from one sample exposed to the HPW environment.

## Conclusion

New tools to monitor the bioburden of high-purity water systems are being developed that rely on laser light scattering and autofluorescence from microbes for detection. Fluorescent microspheres are potential calibration materials for these OWBAs. To provide guidance in choosing parameters for fluorescent microspheres, a relevant bacterial species, *R. pickettii*, that can be found in HPW systems was characterized after exposure to a HPW environment looking at size, count, viability, autofluorescence. When compared with cells exposed to an R2A nutrient environment, the *R. pickettii* exposed to the HPW environment showed lack of growth and smaller sizes. They were, however, still viable and showed a similar, albeit less intense, emission profile upon excitation with 405 nm light, which is commonly used in OWBAs. The fluorescent microspheres were compared and discussed with respect to the bacterial samples, considering size and emission spectra. The emission profiles and intensities from the *R. pickettii* samples and the microsphere populations showed similar spectral coverage at wavelengths greater than ≈ 510 nm with relevant emission intensity. Opportunities exist to expand upon this study to provide further information relevant to identifying applicable and relevant calibration materials for OWBAs.

## Electronic supplementary material

Below is the link to the electronic supplementary material.

Supplementary file1 (PDF 225 kb)
