# Supplementary material for: Evaluating changes to Ralstonia pickettii in high-purity water to guide selection of potential calibration materials for online water bioburden analyzers
Source: J Ind Microbiol Biotechnol. 2019 Jul 25;46(11):1469–78. doi: 10.1007/s10295-019-02192-4 (PMC6826051; doi:10.1007/s10295-019-02192-4)
Supplement: Supplementary file 1 — Supplementary file1 (PDF 225 kb) [file 10295_2019_2192_MOESM1_ESM.pdf]

Supplemental Material

Evaluating changes to *Ralstonia pickettii* in high-purity water to guide selection of potential calibration materials for online water bioburden analyzers

K.D. Benkstein, S.M. Da Silva, N.J. Lin, and D.C. Ripple

DOI :10.1007/s10295-019-02192-4.

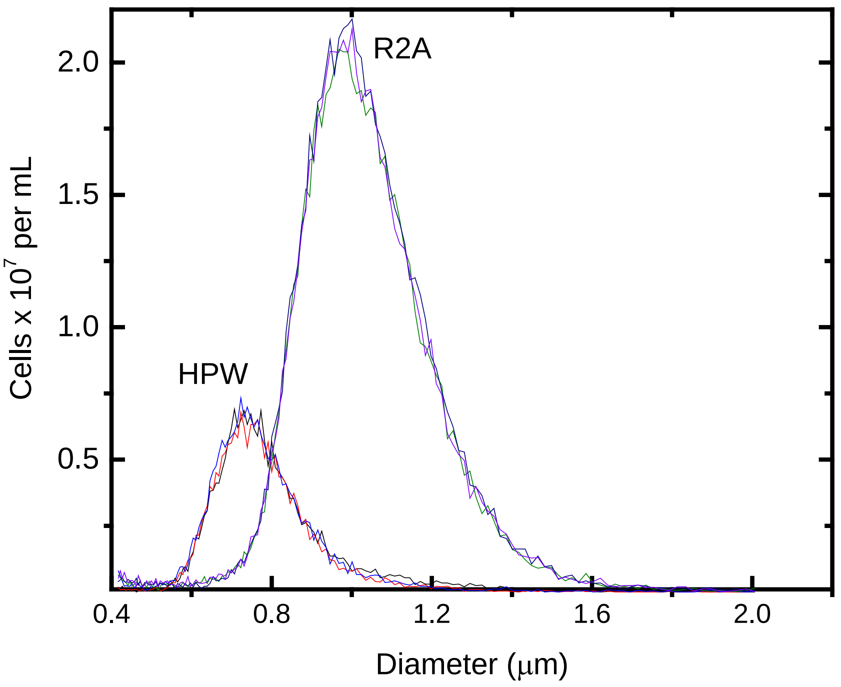

Figure S1: Example distributions of volumetric particle diameter (μm) for *R. pickettii* exposed to HPW and R2A.

Note the shift in particle size when cells are exposed to HPW. For each condition $n = 3$.

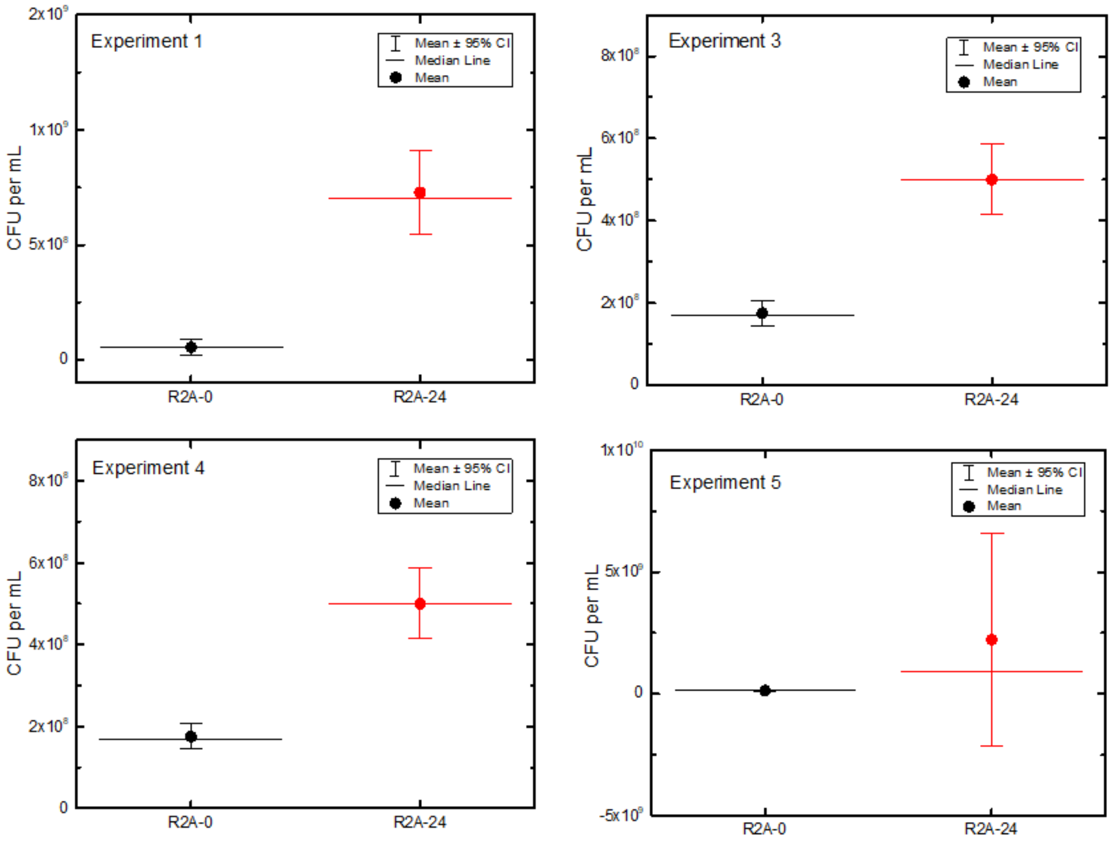

Figure S2. Viability of *R. pickettii* exposed to R2A at two-time points, zero and 24 h. Per day of experiment, the data points varied from ($4 \leq n \leq 9$). The data suggest cell growth in 24 h as expected. Pairwise t-test indicates statistically significant growth for all cases, except for experiment 5.

Statistical analysis pertinent to Figure 4, Figure S2

The results in Figure 4 indicate that the cells remained viable after 24 h in HPW. Statistically significant differences ($P < 0.05$ for Pairwise t-Test) in viability between time $t = 0$ and $t = 24$ h were observed for experiment 1 ($P = 0.0165$). For that day, there was a reduction in cell viability < 3.4-fold. No statistically significant differences were observed for the other days, noting that in case of day 2, the obtained P value is very close to the $P = 0.05$ limit chosen for statistical significance. Overall, an increase of < 1.6-fold in CFU/mL was observed in 3 out of 5 repeat experiments while a decrease in viability (< 3.4-fold) was observed in 2 experiments. In all cases an increase in number of viable cells of < 1.6-fold was observed. For the R2A environment, Figure S2, statistically significant growth was observed in all cases, except for experiment 5.

Effects of scattering on autofluorescence intensities

To check for the effect on intensity from scattering in the samples, spectra were acquired of silica particles ($d \approx$ 1.2 µm) at a concentration of $\approx 1 \times 10^8$ 1/mL. (The refractive index of silica particles, ≈ 1.42 [8], is close to that of bacteria.) Comparing with a similar concentration of *R. pickettii* from the R2A environment, intensity owing to scattering from the silica particles over the integrated range (see Discussion) was, on average, ≈ 6 % of the total emission intensity. A parallel test was also run comparing scattering effects of 1×10^8 1/mL silica particles, $d \approx 0.5$ µm, with autofluorescence from *R. pickettii* exposed to the HPW environment. In that case, the intensity owing to scattering from the silica particles was, on average, ≈ 4 % of the total emission intensity.

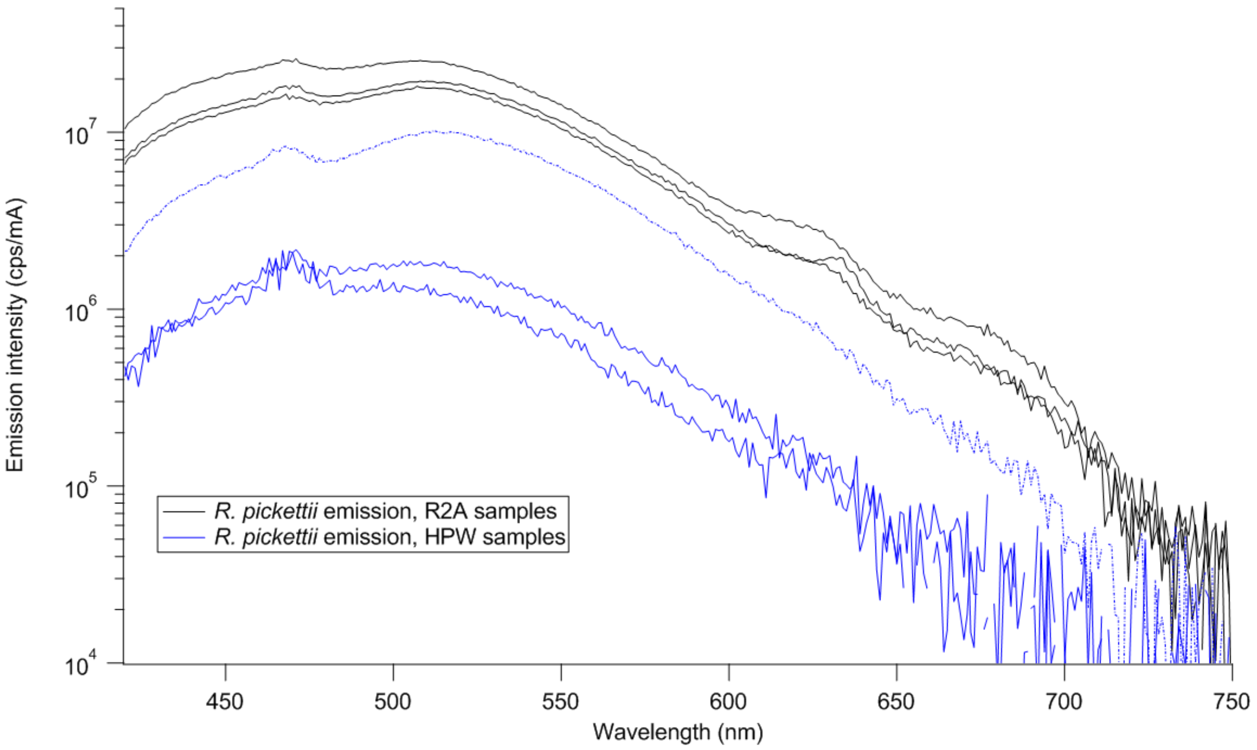

Figure S3. Baseline-subtracted, averaged (n = 3) emission spectra from *R. pickettii* showing three each of samples from R2A environments and HPW environments. Note the unusually high intensity for the most intense HPW sample (dashed spectrum, corresponds to the outlier points in Figure 5), and the lack of an additional peak/shoulder in the range from 600 nm to 650 nm for that spectrum.
